# Lesser Metatarsals Load after Minimally-Invasive Surgery for Hallux Valgus Correction: A Finite Element Model

**DOI:** 10.1055/s-0045-1812999

**Published:** 2025-12-10

**Authors:** Henrique Mansur, Bruno Abdo, Gabriel Ferraz Ferreira, Miguel Viana Pereira Filho, Roberto Zambelli, Gustavo Araújo Nunes

**Affiliations:** 1 Department of Foot and Ankle Surgery, Instituto Return to Play and Instituto D'Or de Pesquisa e Ensino (IDOR), Brasília, DF, Brazil; 2 Department of Foot and Ankle Surgery, Instituto D'Or de Pesquisa e Ensino (IDOR), Brasília, DF, Brazil; 3 Foot and Ankle Surgery Group, Orthopedics and Traumatology Unit, Prevent Senior, São Paulo, SP, Brazil; 4 Head of Foot and Ankle Surgery Group, Orthopedics and Traumatology Unit, Prevent Senior, São Paulo, SP, Brazil; 5 Faculdade de Ciências Médicas de Minas Gerais, Belo Horizonte, MG, Brazil; 6 Rede de Saúde Mater Dei, Belo Horizonte, MG, Brazil; 7 Foot and Ankle Unit, Clínica Ortopédica ÓRION, Brasília, DF, Brazil

**Keywords:** finite element analysis, foot deformities, congenital, hallux valgus, minimally invasive surgical procedures, análise de elementos finitos, deformidades congênitas do pé, hallux valgus, procedimentos cirúrgicos minimamente invasivos

## Abstract

**Objective:**

To analyze the biomechanical consequences on the lesser metatarsals using different screw configurations for fixation of the minimally-invasive Chevron-Akin (MICA) osteotomy, through the finite element method (FEM).

**Methods:**

A FEM model was developed from a computed tomography scan of a moderate hallux valgus (HV) deformity. Five different screw configurations were tested. We measured the maximal tension in the lesser metatarsals for each screw configurations, in physiological and supraphysiological loads.

**Results:**

The lesser metatarsals received the lowest loads when the first metatarsal osteotomy was fixed with one intramedullary and one bicortical screw, with tensile load values varying between 30 and 70 MPa in physiological loads, and 50 to 350 MPa in supraphysiological loads. In all fixing techniques, the 2nd and 4th metatarsals received the highest loads, especially in groups 3 (two bicortical screws) and 5 (only one bicortical screw), with values reaching up to 230 and 600 MPa in physiological and supraphysiological loads, respectively. Regardless of the fixation technique, the region of the lesser metatarsals that received the most load was the diaphysis.

**Conclusion:**

After MICA surgery to correct HV, there is an increase in tension forces on the lesser metatarsals, especially the second and fourth. The technique of fixing the first metatarsal with one bicortical and one intramedullary screw showed the lowest values on the lesser metatarsals load. Furthermore, for physiological and supraphysiological loads, independently of the technique, the forces were concentrated mainly on the metatarsal shaft.

## Resumo

**Objetivo:**

Analisar as consequências biomecânicas nos metatarsos menores do uso de diferentes configurações de parafusos para fixação da osteotomia minimamente invasiva em Chevron e Akin (MICA, do inglês *minimally-invasive Chevron-Akin* ) por meio do método dos elementos finitos (MEF).

**Métodos:**

Um modelo de MEF foi desenvolvido a partir de uma tomografia computadorizada de uma deformidade hallux valgus (HV) moderada. Cinco configurações diferentes de parafusos foram testadas. A tensão máxima nos metatarsos menores de cada configuração de parafuso, em cargas fisiológicas e suprafisiológicas, foi medida.

**Resultados:**

Os metatarsos menores receberam as menores cargas quando a osteotomia do primeiro metatarso foi fixada com um parafuso intramedular e um bicortical, com carga de tração entre 30 e 70 MPa em cargas fisiológicas e 50 a 350 MPa em cargas suprafisiológicas. Em todas as técnicas de fixação, o 2 ^o^ e o 4 ^o^ metatarsos receberam as maiores cargas, especialmente nos grupos 3 (2 parafusos bicorticais) e 5 (apenas 1 parafuso bicortical), com valores de até 230 e 600 MPa em cargas fisiológicas e suprafisiológicas, respectivamente. Independentemente da técnica de fixação, a região dos metatarsos menores submetida à maior carga foi a diáfise.

**Conclusão:**

Após a cirurgia MICA para correção de HV, houve aumento das forças de tensão nos metatarsos menores, especialmente no segundo e quarto. A técnica de fixação do primeiro metatarso com um parafuso bicortical e um intramedular apresentou os menores valores de carga nos metatarsos menores. Além disso, em cargas fisiológicas e suprafisiológicas, independentemente da técnica, as forças concentraram-se principalmente na diáfise metatársica.

## Introduction

The term hallux valgus (HV) refers to a complex three-dimensional (3D) deformity, which consists of medial deviation of the first metatarsal and lateral deviation of the hallux. Although its etiology is multifactorial and not yet fully understood, this is a very common pathology in the population, especially in women. 1 These deformities in the first ray can lead to several changes in the gait biomechanics and mechanical overload on the forefoot, depending on HV severity. 2 3

The definitive treatment of HV is surgical. However, there are several techniques described in the literature. More recently, minimally invasive techniques have been gaining popularity due to their potential for deformity correction and low morbidity, faster recovery, and lower cost. 1

Several studies have highlighted the outcomes of the third generation minimally-invasive Chevron-Akin (MICA) technique for correcting HV. 4 It is known that after surgery, one of the possible complications is the transfer metatarsalgia, caused mainly by excessive shortening and insufficiency of the first metatarsal. 5 6 Another concern is the choice of first metatarsal fixation type and its biomechanical influence on the foot. Although the classical MICA fixation uses two screws (one proximal bicortical and one distal and intramedullary), some authors described modifications using only one screw. 7 8 However, few studies investigated the load in the forefoot after HV correction using this technique. 9 10

The finite element model (FEM) has been used to evaluate the biomechanics of the foot and ankle in various situations. Through validated predefinitions, 11 we can simulate pathologies or surgical procedures, and thus evaluate biomechanical results effectively. 12 A previous study 13 using FEM analysis demonstrated that, after Chevron osteotomy in the first metatarsal, the first ray received less pressure with deviations of 2 to 4 mm and increased pressure with a deviation of 6 mm. Meanwhile, the second ray received less pressure in all degrees of translation, and the other metatarsals received greater pressure, regardless of the first metatarsal's degree of translation. Despite evaluating different degrees of osteotomy translation, they did not analyze the behavior with different fixation techniques of the first metatarsal. 13

The objective of this study is to analyze the biomechanical consequences on the lesser metatarsals after MICA surgery to correct HV, with different techniques for fixation of the osteotomy, through FEM.

## Methods

### Dimensional Characteristics and Screw Insertion Technique

**Fig. 1:**
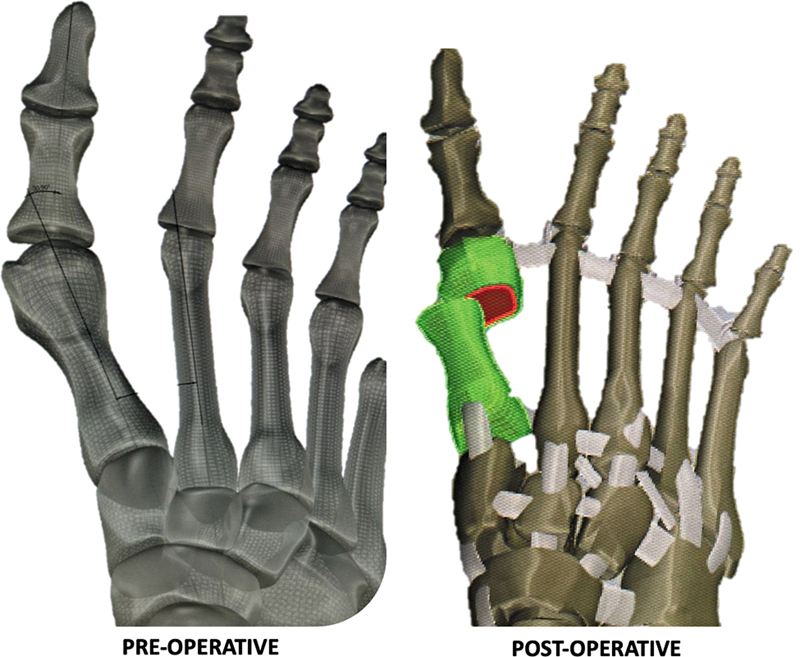
Graphical representation of the pre- and postoperative simulated model for the finite element analysis. Source: Lewis et al. 10

The implants were applied as indicated by the manufacturer's dimensional characteristics (Novastep). The MICA osteotomy was performed at the base of the flare of the distal metaphysis/neck of the first metatarsal, according to Redfern and Vernois's original description of the technique. 14 The head of the first metatarsal was translated laterally 75%, achieving a 5° HV angle (HVA) and a 4° intermetatarsal angle (IMA), as shown in Fig. 1 .

**Fig. 2:**
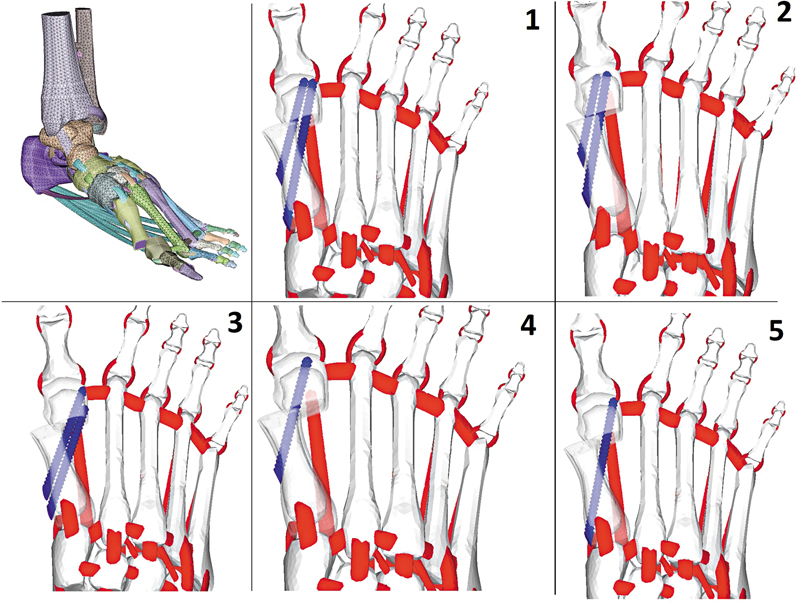
Screw fixation configuration tested by the finite element analysis model: group 1, one bicortical and one intramedullary screw (MICA); group 2, two intramedullary screws; g roup 3 two bicortical screws; group 4, one intramedullary screw; and group 5, one cortical screw. Source: Lewis et al. 10 .

Five groups were categorized based on the technique used for the fixation of the MICA osteotomy. In group 1, named MICA, we fixed the osteotomy with two screws, one bicortical and one monocortical (intramedullary); in 2, two intramedullary screws; in 3, two bicortical screws; in 4, only one intramedullary screw; and in group 5, only one bicortical screw ( Fig. 2 ).

### Biocad Preparation

The program Rhinoceros 6 (Robert McNeel & Associates) created the 3D virtual models of each system (bone and screws). The finite element analysis was performed by the program SimLab (HyperWorks), applying the Optistruct solver (Altair Engineering Inc.). The computed tomography (CT) scans were obtained from the left foot of a 46-years-old female, with a moderate HV deformity (HVA: 30°; and IMA: 14°), without other deformities. The two-dimensional images obtained through CT were used for the 3D reconstruction of the anatomical structure of the surface geometry of the foot by the InVesalius (Centro de Tecnologia da Informação Renato Archer) and STereo Lithography (STL, 3D Systems Inc.) softwares. The CT scan machine used was Emotion (16 channels, Siemens Healthineers) with a slice interval of 2 mm. The Considerations for Reporting Finite Element Analysis Studies in Biomechanics guidelines were followed. 15

### Simulation

To simulate the loading of the metatarsals, FEM was used following fixation of the MICA with five different techniques. First, the files were imported into the SimLab software and each part of the digital models was identified, ensuring that the size of the element was maintained to avoid any issues of contact between the different parts during the simulations.

The discretization of the geometric domain was performed using second-order tetrahedral elements with an average edge length of 3 mm in the cortical and trabecular bones, 0.5 mm in the area, 2 mm in the ligaments, and refinement in the contact regions with an average edge size of 0.8 mm. All tissues were defined as homogeneous, isotropic, and linearly elastic. A tetrahedral element was adopted to form the meshes. The properties of the materials used for simulations were Young's modulus and Poisson's coefficient, following a previous study. 1 A standard mesh sensitivity analysis was performed to ensure that the density used in the FEM was sufficient to reach the converged numerical results and that no further refinement was necessary.

### Boundary and Load Conditions

Considering the physiological conditions, with the forefoot and hindfoot fixed, a vertical ground reaction force (GRF) was applied to the midfoot. No load has been applied on the X and Y-axis. The upward vertical force of the Achilles tendon was also created with half of the value of the GRF. All models were tested for two different physiological conditions (150 and 300 N). The FEM model was applied to measure the maximal tension in each of the lesser metatarsals.

## Results

**Fig. 3:**
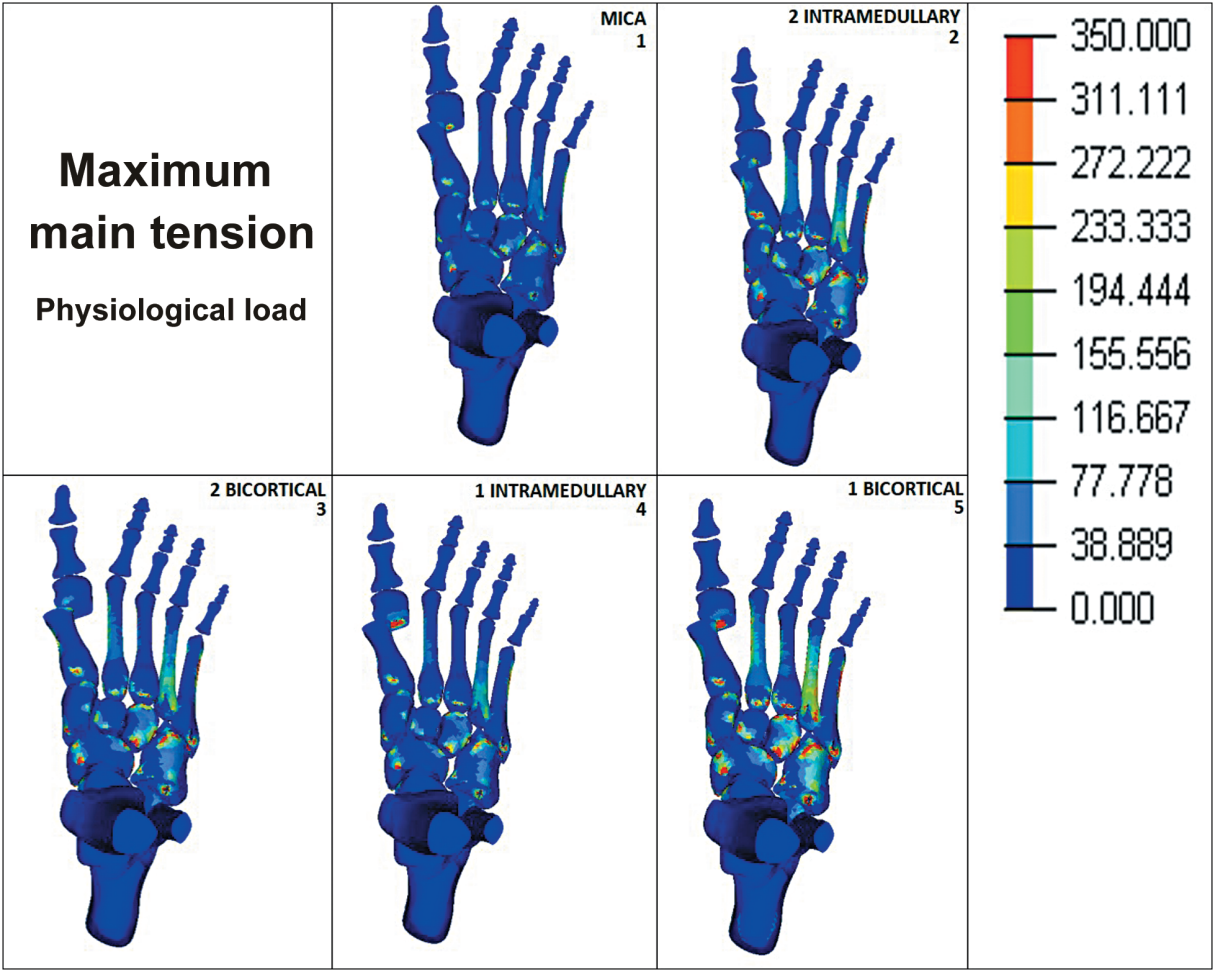
Maximum main tension in physiological load (150 N) in 5 different fixation techniques.

It was observed that when subjected to physiological load, the different methods for fixing the first metatarsal osteotomy presented varied maximum main tension (traction forces) in the lesser metatarsals. Group 1 presented the lowest values, ranging from around 30 to 70 MPa. The highest values were seen in the second and fourth metatarsals in groups 2, 3, and, mainly, in group 5, whose values were around 150 MPa in the second metatarsal and 230 MPa in the fourth. In group 4, the second and fourth metatarsals presented maximum tension of roughly 70 and 115 MPa, respectively ( Fig. 3 ).

**Fig. 4:**
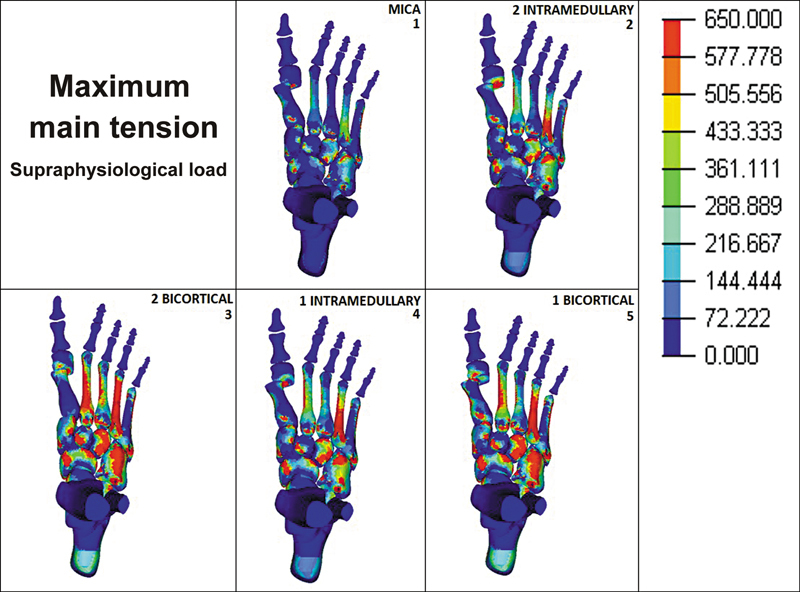
Maximum main tension in supraphysiological load (300 N) in 5 different fixation techniques.

When the MEF models were submitted to supraphysiological load, it was observed that the maximum tension presented higher values in the lesser metatarsals, mainly in the second and fourth. Groups 3 and 5 presented values of around 600 MPa in these metatarsals. In groups 2 and 4, those same metatarsals presented maximum tension of around 300 and 600 MPa, respectively. In group 1 (MICA), the lesser metatarsals presented the lowest maximum tension values, ranging from around 50 to 350 MPa ( Fig. 4 ).

Lastly, it was observed that, in both physiological and supraphysiological loads, the diaphysis was the region of the lesser metatarsals that received the greatest concentration of loads, regardless of the technique used for fixation of the osteotomy of the first metatarsal.

## Discussion

In the present study, we assessed, through the FEM, the metatarsals load after MICA technique to correct HV with different techniques for fixation of the osteotomy. The main findings were the lowest values presented in the lesser metatarsals for group 1 for physiological and supraphysiological loads. Furthermore, for both loads, all other fixation techniques presented the highest values in the second and fourth metatarsals, especially in groups 3 and 5. These results confirm the relevance of using intramedullary screws, together with bicortical ones, which might avoid lesser metatarsal overload.

Minimally-invasive Chevron-Akin osteotomy is a technique used in HV correction that has promising results, with good radiological and clinical outcomes. 4 A prospective study 9 including 31 feet of 25 patients with moderate and severe HV, without metatarsalgia, used pedography to evaluate the load transfers to the lesser metatarsals after surgical correction of HV, with the fourth generation minimally invasive technique. The study demonstrated a reduction in loads on the first ray, with a decrease in central metatarsal loading, 3 months after the surgery. The authors concluded that the technique may not prevent or correct metatarsalgia. Differently, in our study, the MICA technique for correction of HV presented high loads on the lesser metatarsals, especially on the second and fourth.

Previous studies investigated the fixation of the first metatarsal after MICA osteotomy with just one bicortical screw, showing good clinical and radiological results. 7 8 However, none of these studies analyzed load transfer to the lesser metatarsals, postoperatively. In our study, the use of one or two intramedullary (groups 2 and 4) or bicortical (groups 3 and 5) screws showed similar results, with higher loads on the second and fourth metatarsals. Such load transfers to the lesser metatarsals were not observed in the MICA technique, which presented the best biomechanical results. This suggests that adding a second screw with the same position (i.e. intramedullary or bicortical) did not prove to be advantageous in relation to lesser metatarsals' load transfer. Therefore, the use of two screws with different positioning is ideal, according to the MICA technique.

Several complications were described after the first metatarsal osteotomy for HV deformity correction. One of them is transfer metatarsalgia, with an estimated occurrence of 5.4% in percutaneous surgeries. 16 One of its likely causes would be insufficiency of the first ray, either due to excessive shortening or fixation in a dorsiflexion position. 5 6 17 In our study, once the osteotomy was constructed by FEM, there was no deviation on the sagittal plane, nor shortening. That fact could explain increased tension on the lesser metatarsals. Furthermore, curiously, in all fixation types the loads were concentrated more on the lesser metatarsal diaphysis, which may result in transfer metatarsalgia, stress fracture, or toe deformities (claw toes). Therefore, surgeons must be attentive with excessive shortening and deviations of the first metatarsal head in the sagittal plane, avoiding possible overloads on metatarsals.

We are aware of several limitations of our study, most of them related to the FEM analysis. First, not all readers are familiar with this analysis tool. Also, we considered the anatomy from a single foot, only a 75% translation of the first metatarsal head with one model loading type. Further, for modeling purposes, it was considered that the mechanical properties of the materials, cortical bone, trabecular bone, ligaments, and syntheses were continuous, isotropic, and uniform linear elastic materials.

The accuracy of FEM results depends on the input parameters and assumptions made during model development. As such, inaccurate material properties can lead to divergent results. Simplifications in modeling, such as assuming linear material behavior or limited anatomy, may affect the accuracy of predictions. Furthermore, it is important to highlight that different results may arise in a dynamic simulation, different from the static simulation performed in this study. Also, it's impossible to consider the interindividual variability and other in vivo compensatory mechanisms. This model also could not account for different osteotomy configurations or percentage metatarsal head shifts. Therefore, the results presented here may vary from in vivo studies. However, as our objective was to evaluate only the fixation methods, we tried to recreate a test excluding the variations found in human or cadaveric studies.

## Conclusion

In the present study, FEM analysis showed that after MICA osteotomy for HV correction, there is an increase in tension forces on the lesser metatarsals, especially the second and fourth. The technique fixing the first metatarsal with one bicortical and one intramedullary screw showed the lowest values in the lesser metatarsals loads. Besides, for physiological and supraphysiological loads, the forces were concentrated mainly in the metatarsal shaft independent of the technique.
